# Supplementary material for: Caudal–Rostral Progression of Alpha Motoneuron Degeneration in the SOD1G93A Mouse Model of Amyotrophic Lateral Sclerosis
Source: Antioxidants (Basel). 2022 May 17;11(5):983. doi: 10.3390/antiox11050983 (PMC9137889; doi:10.3390/antiox11050983)
Supplement: Supplementary file 1 [file antioxidants-11-00983-s001.zip › antioxidants-1700618-supplementary.pdf]

Supplementary Materials

Table S1. Size comparison of α-MNs and γ-MNs between SOD1^{G93A} and WT mice:.

Group	Statistical test	N	P Values
SOD1^{G93A} v WT (α-MNs v γ-MNs)	Two-Way ANOVA, Tukey's multiple comparison test	SOD1^{G93A}: α-MNs (4) γ-MNs (4) WT: α-MNs (4) γ-MNs (4)	Genotype: 0.0015 MN classification: <0.001 Interaction: 0.0087
Distribution analysis: α-MNs: SOD1^{G93A} v WT	Mann Whitney U Test.	SOD1^{G93A+}: α-MNs (60) WT: α-MNs (60)	P <0.001 (Two tailed)
Distribution analysis: γ -MNs: SOD1^{G93A} v WT	Mann Whitney U Test.	SOD1^{G93A}: γ-MNs (4) WT: γ-MNs (4)	P <0.001 (Two tailed)
Distribution analysis: αp-MNs v WT α-MNs	Mann Whitney U Test.	αp-MNs (22) WT: α-MNs (38)	P 0.912 (Two tailed)

Table S2. MNs loss in between 3- and 4- months in SOD1^{G93A} and WT mice: (* Indicates significance.).

Group	Statistical test	N	P Values
**α-MNs Lumbar** SOD1^{G93A} v WT (3-Months v 4-Months)	Two-Way ANOVA, Tukey's multiple comparison test	3- Month: WT (5), SOD1^{G93A} (3), 4- Month: WT (3), SOD1^{G93A} (5).	*Genotype: 0.0009 Age: 0.8368 Interaction: 0.8929
**γ-MNs Lumbar** SOD1^{G93A} v WT (3-Months v 4-Months)	Two-Way ANOVA, Tukey's multiple comparison test	3- Month: WT (5), SOD1^{G93A} (3), 4- Month: WT (3), SOD1^{G93A} (5).	Genotype: 0.508 Age: 0.705 Interaction: 0.07
**α-MNs Cervical** SOD1^{G93A} v WT (3-Months v 4-Months)	Two-Way ANOVA, Tukey's multiple comparison test	3- Month: WT (5), SOD1^{G93A} (3), 4- Month: WT (3), SOD1^{G93A} (5).	*Genotype: 0.0002 Age: 0.2472 Interaction: 0.6382
**γ-MNs Cervical** SOD1^{G93A} v WT (3-Months v 4-Months)	Two-Way ANOVA, Tukey's multiple comparison test	3- Month: WT (5), SOD1^{G93A} (3), 4- Month: WT (3), SOD1^{G93A} (5).	Genotype: 0.9457 Age: 0.5898 Interaction: 0.7307
**WT α-MNs** Lumbar v Cervical (3-Months v 4-Months)	Two-Way ANOVA, Tukey's multiple comparison test	3- Month: Lumbar (5), Cervical (5), 4- Month: Lumbar (3), Cervical (3).	Spinal level: 0.2139 Age: 0.3193 Interaction: 0.9633
**SOD1^{G93A} α-MNs** Lumbar v Cervical (3-Months v 4-Months)	Two-Way ANOVA, Tukey's multiple comparison test	3- Month: Lumbar (5), Cervical (5), 4- Month: Lumbar (3), Cervical (3).	*Spinal level: 0.002 Age: 0.205 *Interaction: 0.0415
**WT γ-MNs** Lumbar v Cervical (3-Months v 4-Months)	Two-Way ANOVA, Tukey's multiple comparison test	3- Month: Lumbar (5), Cervical (5), 4- Month: Lumbar (3), Cervical (3).	Spinal level: 0.0022 Age: 0.9420 Interaction: 0.2664
**SOD1^{G93A} γ-MNs** Lumbar v Cervical (3-Months v 4-Months)	Two-Way ANOVA, Tukey's multiple comparison test	3- Month: Lumbar (5), Cervical (5), 4- Month: Lumbar (3), Cervical (3).	Spinal level: 0.2764 Age: 0.4078 Interaction: 0.2807

Table S3. α-MNs counts in the phrenic nucleus between 3 and 4 months.

Group	Statistical test	N	P Values
**α-MNs Phrenic nucleus** SOD1^{G93A} v WT 3-Months v 4-Months	Two-Way ANOVA, Tukey's multiple comparison test	3- Month: WT (5), SOD1^{G93A} (3). 4- Month: WT (3), SOD1^{G93A} (5).	*Genotype: 0.001 Age: 0.0312 Interaction: 0.8269

Table S4. PKC-y intensity between 3 and 4 months in SOD1 and WT mice:.

Group	Statistical test	N	P Values
**Lumbar** SOD1^{G93A} v WT (3-Months v 4-Months)	Two-Way ANOVA, Tukey's multiple comparison test	3-Month: WT (5), SOD1^{G93A} (4), 4-Month: WT (3), SOD1^{G93A} (3).	Genotype: 0.0521 Age: 0.0786 Interaction: 0.6485
**Cervical** SOD1^{G93A} v WT (3-Months v 4-Months)	Two-Way ANOVA, Tukey's multiple comparison test	3-Month: WT (5), SOD1^{G93A} (3), 4-Month: WT (3), SOD1^{G93A} (3).	Genotype: 0.3866 Age: 0.7955 Interaction: 0.4244
